# Demonstration program of population-based lung cancer screening in China: Rationale and study design

**DOI:** 10.1111/1759-7714.12078

**Published:** 2014-04-22

**Authors:** Qinghua Zhou, Yaguang Fan, Ning Wu, Yunchao Huang, Ying Wang, Lu Li, Jiewei Liu, Xinyun Wang, Weimin Li, Youlin Qiao

**Affiliations:** 1 Tianjin Key Laboratory of Lung Cancer Metastasis and Tumor Environment, Tianjin Lung Cancer Institute, Tianjin Medical University General HospitalTianjin, China; 2 Department of Diagnostic Radiology, Chinese Academy of Medical Sciences, Peking Union Medical College, Cancer HospitalBeijing, China; 3 Department of Thoracic Surgery, the Third Affiliated Hospital of Kunming Medical College (Yunnan Tumor Hospital)Kunming, Yunnan, China; 4 Department of Radiology, Tianjin Medical University General HospitalTianjin, China; 5 Medical Oncology, Cancer Center, West China Hospital, Sichuan UniversityChengdu, Sichuan, China; 6 Department of Pathology, Tianjin Medical University General HospitalTianjin, China; 7 Department of Respiratory Medicine, West China Hospital, Sichuan UniversityChengdu, Sichuan, China; 8 Department of Cancer Epidemiology, Cancer Institute, Chinese Academy of Medical Sciences and Peking Union Medical CollegeBeijing, China

**Keywords:** Feasibility study, lung cancer, mass screening, spiral computed tomography

## Abstract

**Background:**

Lung cancer is the leading cause of cancer-related death and has become an enormous economic burden in China. Low-dose spiral computed tomography (LDCT) screening could reduce lung cancer mortality. The feasibility of conducting a population-based lung cancer screening with LDCT in China is uncertain.

**Methods:**

In 2010, a demonstration program of lung cancer screening was initiated in China. High-risk individuals were enrolled in a cluster sampling design in different centers. Participants received baseline and annual screening with spiral CT and follow-up information was collected. The objective of this program is to evaluate the feasibility of conducting population-based LDCT lung cancer screening in the Chinese context. The rates of detection, early diagnosis and treatment are defined as indicators of program performance. The optimal management strategies for nodules are explored in the Chinese context based on experiences in other studies overseas.

**Results:**

A demonstration program of ongoing prospective, multi-center, population-based lung cancer screening is being performed in China.

**Conclusions:**

This demonstration program will provide opportunities to explore the feasibility of LDCT lung cancer screening in the Chinese setting.

## Introduction

Lung cancer is the leading cause of cancer-related death worldwide.^1^In China, according to Third National Death Survey conducted in 2004–2005, lung cancer mortality has increased 465% during the past 30 years.^2^Lung cancer has become an enormous economic burden in China. Based on data in the China Statistical Yearbook, it was estimated that the total inpatient cost increased from $2.16 billion to $6.33 billion from 1999 to 2005, with an average annual increase of 16.15%.^3^

Several risk factors, including smoking, involuntary smoking, occupational exposure, indoor radon, genetics, and previous lung disease contribute to lung cancer death.^4^–^11^Among these, smoking is the major cause of lung cancer, accounting for 75.04% of male and 18.35% of female lung cancer deaths in China.^12^Though smoking control is the most effective measure for the primary prevention of lung cancer, an upward trend of lung cancer incidence and mortality is still expected in future decades in China because of the high prevalence of smoking.

Lung cancer survival is closely related to the stage at diagnosis, that is, its prognosis is more favorable when diagnosed at an earlier stage. This provided a rationale for lung cancer screening. Since the 1960s, several randomized controlled trials (RCTs) of lung cancer screening with chest radiograph and/or sputum cytology have been conducted, but have shown no benefit in a reduction to the mortality of lung cancer.^13^–^15^These results were confirmed by the Prostate, Lung, Colorectal and Ovarian (PLCO) Cancer Screening Program.^16^Compared with chest radiograph, low dose spiral computed tomography (LDCT) is three to four times more sensitive to lung cancer detection, and lung cancers detected by LDCT had better stage distribution and patient rates of survival.^17^,^18^Most importantly, results of the National Lung Cancer Screening Program (NLST) have demonstrated a 20% reduced lung cancer mortality with LDCT among heavy smokers.^19^Based on this result, some medical organizations have revised the guidelines for lung cancer screening.^20^–^23^In 2010, a demonstration program of population-based lung cancer screening was initiated in China; the objective of this program is to evaluate the feasibility of conducting population-based LDCT lung cancer screening in the Chinese context.

## Materials and methods

### Overall design

Initiated in 2010, this demonstration program is an ongoing prospective, multi-center observational study of screening with LDCT. During 2010 to 2013, there were two study centers: Dagang Oilfield in Tianjin and Xuanwei City in Yunnan Province. The number of centers will be expanded and cover several regions including Beijing City and Sichuan Province in 2013 and 2014, respectively. In each center, participants with high lung cancer risk are enrolled based on a cluster sampling design.

In this program, the rates of detection, early diagnosis and treatment are defined as indicators of program performance. Optimal management strategies for nodules and healthcare utilization are explored in the Chinese context based on the results of other studies overseas. In addition, we will investigate the effect of screening on smoking cessation. If possible, we will search and validate the biomarkers of early lung cancer in the screening cohort and evaluate whether early lung cancer biomarkers can augment LDCT accuracy and reduce the aftereffect of high false positive LDCT results.

## Methods

### Organization and implementation of the program

**Figure 1:**
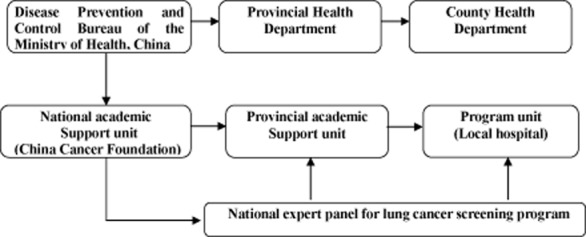
Organization and implementation of lung cancer screening program.

The administration of this program includes three levels: the *Cancer Foundation*of *China*, on behalf of the Disease Prevention and Control Bureau of the Ministry of Health; the Provincial Department of Public Health; and the County Department of Public Health. The program is conducted in local hospitals at county level and national and provincial experts provide academic support. Figure 1illustrates the organization and implementation of the program. The Cancer Foundation of China Institutional Review Board approved this study.

### Participants

In order to ensure efficiency, lung cancer screening is conducted among high-risk populations. In this program, the criteria for high-risk population are defined according to smoking history, age distribution, and other risk factors in different regions and centers. For example, at the Dagang Oilfield center, participants are staff aged between 50–74 and with at least 20 pack-years smoking history, while at the Xuanwei center, indoor air pollution is also considered as inclusion criteria because of the high incidence of lung cancer in females, mainly caused by domestic coal use.^24^Individuals who have a history of cancer within the last five years (with the exception of non-melanoma skin cancer, cervical carcinoma in situ, and localized prostate cancer), cannot tolerate possible lung cancer resection, or with life threatening disease are excluded from this program.

### Screening procedure

#### Informed consent

All participants are required to attend an informed consent meeting. Program staff explain the aim and significance, benefits, and potential hazards of the screening. Staff read the informed consent form and answer participants' questions. All participants sign the informed consent on a voluntary basis.

#### Baseline survey

Participants then fill out an information questionnaire, which includes basic personal information, lifestyle, previous medical history, and family history of cancer. The IDs of participants who followed up are the same at baseline as at follow up, and the period of follow-up time will be stated.

### Low-dose spiral computed tomography (LDCT) scan

Scan parameters: 120kVp; 30mAs; 350 mm scan length; 3.7 seconds scan time; reconstruction slice thicknesses contiguous 0.625–1.25 mm (slice interval is 0), if the conditions are not adequate, a contiguous 5 mm thick reconstruction slice thickness can be adopted. Scan range: from apex pulmonis to costophrenic angle (all lung regions are scanned). The CT scan will be completed at the end of an inhalation within a single breath-hold. Images of contiguous 5 mm axial slices and 0.625–1.25 mm –thickness thin-slice reconstructions are transmitted to the Picture Archiving and Communication System (PACS) or are archived by disc burning.

#### Image observation

Images are read by senior radiologists who examine the standard lung window, the bone window, and the mediastinal window. The width of the lung window is usually 1600HU ∼ 2000HU, and window level is −600HU ∼ –700HU. Two senior radiologists in each center independently view scans, and a provincial expert panel resolves any differences.

#### Nodule measurements

**Figure 2:**
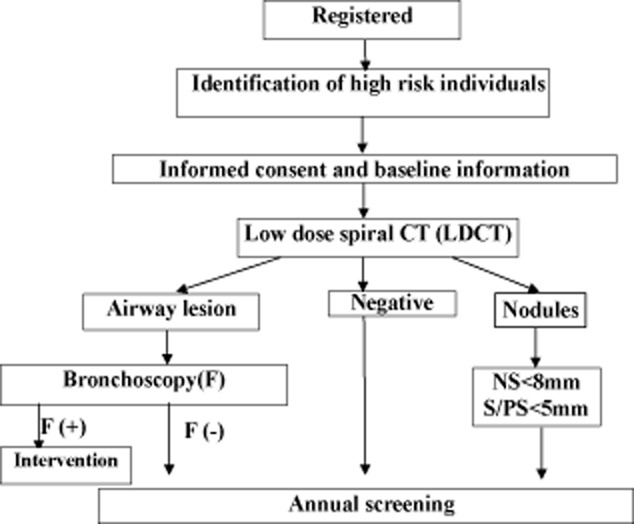
Baseline screening flowchart (1). S (solid nodules); PS (part solid nodules); NS (non solid nodules).

**Figure 3:**
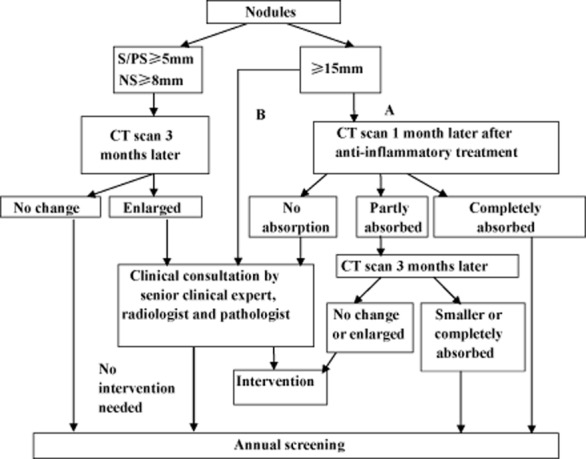
Baseline screening flowchart (2). S (solid nodules); PS (part solid nodules); NS (non solid nodules).

**Figure 4:**
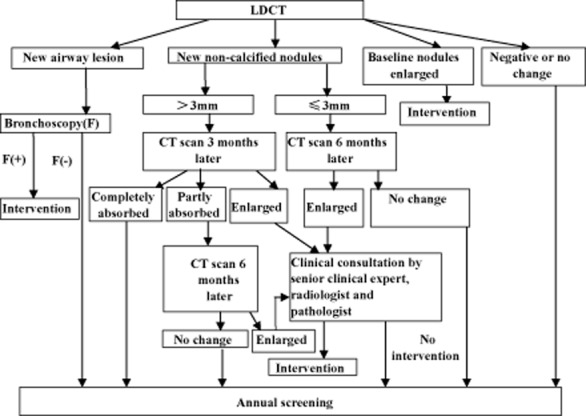
Annual screening flow chart.

The length and width diameters are measured by electronic ruler at the maximum cross-section (note: length diameter refers to the maximum diameter of the largest cross-section, and width diameter refers to the diameter perpendicular to the maximum diameter). The high-risk population receives LDCT at baseline, and then an annual screening scan. Flowcharts of baseline screening, nodule management in baseline screening, and a flowchart of annual screening are shown in Figures 2–4.

### Nodule management and follow up

Nodules detected by LDCT are classified into two groups: definitely benign or calcified nodules, and uncertain or non-calcified nodules. The latter groups are followed up according to the properties and size of the nodules.

### Nodule detected in baseline screening

Participants with solid or part-solid nodules <5 mm in diameter, or non-solid nodules <8 mm in diameter, participate in annual screening. For those have solid or part-solid nodules 5–14 mm in diameter or non-solid nodules 8–14 mm in diameter, follow-up is by routine CT three months later. If nodules are determined to be growing in size, clinical consultation by a senior clinical expert, a radiologist, and a pathologist is recommended to determine the necessity of clinical intervention. If no growth is detected, participants are recommended for re-screening in the next round. For those with nodules 15 mm or more than 15 mm, two pathways can be selected: immediate clinical consultation, or rescan after anti-inflammatory treatment. For the latter, if the nodules are absorbed, then participants will enter into the next round screening visit, but if no change to the nodules is detected, then clinical consultation is needed. If the nodules are partly absorbed, a further CT scan will be conducted, and based on the changes of nodules, different solutions will be taken.

### Nodule detected in annual screening

For participants with ≤3 mm new nodules, a rescan is recommended after six months; if the nodules have grown, these participants receive clinical consultation to determine the necessity of clinical intervention, or they will receive a screening visit in the next round. For participants with >3 mm new nodules, a rescan is recommended three months later (when necessary, anti-inflammatory treatment is provided in advance), and if nodules have grown, these participants will receive clinical consultation; if the nodules are absorbed completely, then participants wait for the next round of annual screening. However, if nodules are just partly absorbed, a second rescan is provided six months later, and if the nodules have grown, participants will receive clinical consultation, or join the next annual screening. When a significant growth or an increase of the solid components of the nodule is found, compared to earlier scan images, clinical intervention is advised. Bronchoscopy is recommended if suspicious tracheal and/or bronchial lesions are detected.

### Follow up

All participants are followed up for several years to confirm the outcome specific to lung cancer, including screening-detected and interval cases.

### Quality control

A uniform quality control form is designed to confirm that the informed consent form, baseline information questionnaire, LDCT log, and follow-up information are all collected. A LDCT scan should comply with the screening procedure. Suggestions are offered for detected non-calcified nodules. Images of ≥5 mm solid nodules and ≥8 mm non-solid nodules and suspicious lesions are re-examined by a panel of thoracic radiologists and thoracic surgeons.

## Discussion

With the increasing burden of cancer, the Ministry of Health of China issued the “Compendium of Cancer Prevention and Control in China (2004–2010)” in 2003.^25^The compendium addressed that early detection, diagnosis, and treatment of cancer should be carried out according to local conditions. On the basis of this compendium, in 2005, the Ministry of Finance and the Ministry of Health included the cancer early detection and treatment program into a special fund program supported by the Chinese Central Government Public Health Special Subsidy. By the end of 2009, six kinds of cancers (cervical, esophageal, liver, colorectal, nasopharyngeal, and gastric cancer) have been included into this program. However, as the effectiveness of the screening program had not yet been confirmed, lung cancer screening was not included.

By 2009, the excellent prognosis of lung cancer cases detected by LDCT saw lung cancer screening included into the funding program, even though its effectiveness had still not been confirmed at that time. In 2011, the National Lung Screening Trial (NLST) reported a 20% lower lung cancer specific mortality rate in the LDCT-screened arm compared to the chest x-ray (CXR)-screened arm, demonstrating for the first time that screening can reduce lung cancer mortality.^19^However, whether the NLST result cancer can be generalized in the China setting is unclear, because of the differences between disease epidemic, healthcare system, quality of service delivery, and the acceptance or adherence of populations.

The implementation of a population-based screening program is a complex process. It requires a collaborative and multidisciplinary effort and considerable resources in advance. To ensure the sustainable development of a national screening program, three key components must be fulfilled: effective health promotion to increase the acceptance of participants; the expansion of qualified primary health technical teams in local or primary hospitals; and the scheme of lung cancer screening should fit the cost-benefit principle combined with the medical care system in China.^26^Local technical personnel receive periodic technical training and there have been attempts to incorporate this program into the health insurance system in China. This program has now been included into the special program of medical insurance system reform in China.

In order to maximize the potential benefits, lung cancer screening has always been conducted among high-risk populations. The main criteria for a high-risk population in lung cancer screening programs have mainly focused on age and smoking status, and, consequently, many other risk factors are not considered. The National Comprehensive Cancer Network (NCCN) guideline extends screening to other high-risk populations based on the assumption that these are populations with a similar lung cancer risk and will derive a similar benefit from screening.^23^In addition, models have been developed to predict lung cancer risk in lung cancer screening programs.^27^,^28^However, both the NCCN guideline and lung cancer risk-prediction models require further validation and refinement before they can be applied to routine screening programs in different populations. In this program, the criteria for high-risk populations were not defined uniformly, but were based on smoking history, age distribution, and other risk factors in different regions and centers.

One of the disadvantages of LDCT scanning is that it can yield a high rate of false-positive results, which may lead to invasive follow-up testing, increased radiation risk, financial burden, and anxiety. In the NLST program, positive tests accounted for 24.2% of the total number of LDCT screening tests, however, the proportion of false positive results was as high as 96.4%.^19^Thus, more research is needed to better understand the optimal strategy of indeterminate pulmonary nodule management. In our program, we compare the difference in positive rates, false positive rates, and the clinical pathway and work-up of positive patients in European and North American lung cancer screening programs, and explore the optimal nodule management of pulmonary nodules in the Chinese context.^29^For example, we also incorporate the measurement of the volume doubling time of nodules into the management strategy in the Dagang Oilfield center.

Although the effectiveness of LDCT screening has been confirmed, questions remain regarding the definition of high-risk population, high false positive rate, and radiation risk.^30^Molecular markers may be helpful for risk prediction, and in combination with LDCT in lung cancer screening may reduce high false positive results.^31^,^32^In our continuing study, we will perform biomarker studies to precisely predict lung cancer risk, including gene polymorphism, gene methylation, and sputum atypia. In addition, we will collect specimens (blood, sputum) to search for the markers of early lung cancer.

LDCT screening may also provide a good opportunity for the delivery of effective smoking cessation interventions.^33^,^34^The International Association for the Study of Lung Cancer (IASLC) Screening Workshop 2011 recommended the integration of smoking cessation practices into future lung cancer CT screening programs.^35^We will also evaluate the effect of screening-associated smoking cessation methods in our program.

## Conclusion

Lung cancer is now the biggest burden of cancer-related death in China. Under these circumstances and in light of the positive developments of LDCT screening in recent years, China initiated a demonstration program of LDCT lung cancer screening ahead of the results of the NLST program. In consideration of the differences in population, risk factors, and healthcare structures between countries, it is necessary to adapt results from overseas studies to optimize the screening protocol in China. This demonstration program will provide opportunities to explore NLST results in the Chinese setting.

## References

[b1] Ferlay J, Shin HR, Bray F, Forman D, Mathers C, Parkin DM (2008). Estimates of worldwide burden of cancer in GLOBOCAN. Int J Cancer.

[b2] MoHotpsro C (2010). Report of the Third National Retrospective Survey of Death Causes in China.

[b3] Wang MWY, Guo B (2007). Status quo and issues of direct impatient cost of lung carcinoma in China. Chin Health Econ.

[b4] Boffetta P, Agudo A, Ahrens W (1998). Multicenter case-control study of exposure to environmental tobacco smoke and lung cancer in Europe. J Natl Cancer Inst.

[b5] Krewski D, Lubin JH, Zielinski JM (2005). Residential radon and risk of lung cancer: a combined analysis of 7 North American case-control studies. Epidemiology.

[b6] Steenland K, Loomis D, Shy C, Simonsen N (1996). Review of occupational lung carcinogens. Am J Ind Med.

[b7] Brenner DR, McLaughlin JR, Hung RJ (2011). Previous lung diseases and lung cancer risk: a systematic review and meta-analysis. PLoS ONE.

[b8] Yao Y, Fan Y, Wu J (2012). Potential application of non-small cell lung cancer-associated autoantibodies to early cancer diagnosis. Biochem Biophys Res Commun.

[b9] Mei CR, Luo M, Li HM, Deng WJ, Zhou QH (2011). DNA repair gene polymorphisms in the nucleotide excision repair pathway and lung cancer risk: a meta-analysis. Chin J Cancer Res.

[b10] Liu X, Fan Y, Jiang Y (2013). A cohort study on risk factors of lung cancer in Yunnan tin miners]. Zhongguo Fei Ai Za Zhi.

[b11] Gu J, Wen Y, Zhu S (2013). Association between P(16INK4a) promoter methylation and non-small cell lung cancer: a meta-analysis. PLoS ONE.

[b12] Wang J-B, Fan Y-G, Jiang Y (2011). Attributable causes of lung cancer incidence and mortality in China. Thorac Cancer.

[b13] Frost JK, Ball WC Jr LevinML (1984). Early lung cancer detection: results of the initial (prevalence) radiologic and cytologic screening in the Johns Hopkins study. Am Rev Respir Dis.

[b14] Fontana RS, Sanderson DR, Woolner LB, Taylor WF, Miller WE, Muhm JR (1986). Lung cancer screening: the Mayo program. J Occup Med.

[b15] Kubik A, Polák J (1986). Lung cancer detection. Results of a randomized prospective study in Czechoslovakia. Cancer.

[b16] Oken MM, Hocking WG, Kvale PA (2011). Screening by chest radiograph and lung cancer mortality: the Prostate, Lung, Colorectal, and Ovarian (PLCO) randomized trial. JAMA.

[b17] Yau G, Lock M, Rodrigues G (2007). Systematic review of baseline low-dose CT lung cancer screening. Lung Cancer.

[b18] Henschke CI, Yankelevitz DF, International Early Lung Cancer Action Program Investigators (2006). Survival of patients with stage I lung cancer detected on CT screening. N Engl J Med.

[b19] Aberle DR, Adams AM, National Lung Screening Trial Research Team (2011). Reduced lung-cancer mortality with low-dose computed tomographic screening. N Engl J Med.

[b20] Wender R, Fontham ET, Barrera E (2013). American Cancer Society lung cancer screening guidelines. CA Cancer J Clin.

[b21] Jaklitsch MT, Jacobson Fl, Austin JH (2012). The American Association for Thoracic Surgery guidelines for lung cancer screening using low-dose computed tomography scans for lung cancer survivors and other high-risk groups. J Thorac Cardiovasc Surg.

[b22] Detterbeck FC, Mazzone PJ, Naidich DP,BachPB (2013). Screening for lung cancer: diagnosis and management of lung cancer, 3rd ed: American College of Chest Physicians evidence-based clinical practice guidelines. Chest.

[b23] National Comprehensive Cancer Network (2012).

[b24] Barone-Adesi F, Chapman RS, Silverman DT (2012). Risk of lung cancer associated with domestic use of coal in Xuanwei, China: retrospective cohort study. BMJ.

[b25] Ministry of Health PsRoC (2004). Compendium of cancer prevention and control in China (for year 2004-2010). China Cancer.

[b26] Dong Zhi-wei, Qiao You-lin (2009). The practice and discussion of population-based cancer screening program in China. China Cancer.

[b27] Tammemagi CM, Pinsky PF, Caporaso NE (2011). Lung cancer risk prediction: prostate, lung, colorectal and ovarian cancer screening trial models and validation. J Natl Cancer Inst.

[b28] Cassidy A, Myles JP, van Tongeren M (2008). The LLP risk model: an individual risk prediction model for lung cancer. Br J Cancer.

[b29] Nair A, Hansell DM (2011). European and North American lung cancer screening experience and implications for pulmonary nodule management. Eur Radiol.

[b30] Bach PB, Mirkin JN, Oliver TK (2012). Benefits and harms of CT screening for lung cancer: a systematic review. JAMA.

[b31] Reisman DN, Marquez S (2012). Biomarkers can promote risk stratification in CT Scanning for lung cancer detection. J Clin Exp Oncol.

[b32] Dong X, Bicheng Z, Donald D, Kui S, Goetz K, Carl F (2013). Lung cancer screening: from imaging to biomarker. Biomarker Res.

[b33] MacRedmond R, McVey G, Lee M (2006). Screening for lung cancer using low dose CT scanning: results of 2 year follow up. Thorax.

[b34] Poghosyan H, Kennedy Sheldon L, Cooley ME (2012). The impact of computed tomography screening for lung cancer on smoking behaviors: a teachable moment. Cancer Nurs.

[b35] Field JK, Smith RA, Aberle DR (2012). International Association for the Study of Lung Cancer Computed Tomography Screening Workshop 2011 report. J Thorac Oncol.

